# Impact of Heat Stress on Intake, Performance, Digestibility, and Health of Neonatal Dairy Calves

**DOI:** 10.3390/ani15131876

**Published:** 2025-06-25

**Authors:** Luiz F. M. Neves, Mariana B. Gomes, Joana P. Campolina, Mariana M. Campos, Eduardo M. B. Souza, Jaciara Diavão, Abias S. Silva, Thierry R. Tomich, Wanessa A. Carvalho, Helena F. Lage, Sandra G. Coelho

**Affiliations:** 1 Departamento de Zootecnia, Escola de Veterinária, Universidade Federal de Minas Gerais, Belo Horizonte 30161-970, MG, Brazil; luizneveszoo@yahoo.com.br (L.F.M.N.); mariana.brito.gomes@gmail.com (M.B.G.); joana.campolina@yahoo.com.br (J.P.C.); eduardomoreirabarradas@hotmail.com (E.M.B.S.); helenaf.lage@gmail.com (H.F.L.); 2 Brazilian Agricultural Research Corporation—Embrapa Gado de Leite, Juiz de Fora 36038-330, MG, Brazil; mariana.campos@embrapa.br (M.M.C.); jacidiavao@gmail.com (J.D.); abias.severo@gmail.com (A.S.S.); thierry.tomich@embrapa.br (T.R.T.); wanessa.carvalho@embrapa.br (W.A.C.)

**Keywords:** ambiance, dairy calf, discomfort, pre-weaning phase, temperature

## Abstract

This study investigates the effects of heat stress in a climate chamber from day 0 to 28 days of life on physiological responses, intake, nutrient digestibility, immunity, and performance in neonatal Holstein calves. Thirty-four calves (nineteen females, fifteen males) were randomly assigned to a control group (CON, temperature–humidity index [THI] 66, with 22 °C and 65% humidity for 24 h, n = 17) or a heat-stressed (HS) group, which was exposed to a THI of 82, 32 °C, and 65% humidity for 9 h, followed by a THI of 66, 22 °C, and 65% humidity for 15 h. The HS calves exhibited increased respiratory rates and rectal temperatures (*p* < 0.001), particularly during heat exposure periods, as well as a 59.5% increase in water intake compared to CON. While milk and solid feed intake, average daily gain, and feed efficiency were similar between groups, HS calves had reduced ether extract digestibility and altered ruminal fermentation, including lower acetate and lower propionate concentrations. The blood cytokine analysis showed elevated interleukin-4 and reduced interleukin-8 and IP-10 levels in heat-stressed calves, indicating a shift toward an anti-inflammatory immune profile. Despite no major performance impairments, heat stress has induced clear physiological, digestive, and immunological changes. These results underscore the importance of implementing thermal mitigation strategies during early life to safeguard calf health and development in increasingly warmer climates.

## Simple Summary

Rising global temperatures have intensified concerns about heat stress in young dairy calves. This study evaluates the effects of heat stress on physiology, intake, digestion, immunity, and performance of Holstein calves during their first 28 days of life. Calves were housed in a climate chamber and exposed daily to either a thermoneutral environment or 9 h of heat stress. Heat-stressed calves showed increased respiratory rates, rectal temperatures, and water intake. Despite similar growth rates between groups, heat stress reduced the digestibility of dietary fat, altered ruminal fermentation, and shifted cytokine profiles toward a less inflammatory state. These findings highlight that early-life heat exposure can affect calf physiology and metabolism, even without visible changes in performance, emphasizing the importance of environmental management during the neonatal period.

## 1. Introduction

Holstein dairy calves (*Bos taurus taurus*) are often perceived as less susceptible to heat stress compared to dairy cows, due to lower metabolic heat production and greater efficiency in heat dissipation. However, young animals can still be negatively impacted by rising environmental temperatures [1]. Heat stress in dairy calves is typically induced when temperatures exceed 26 °C [2]. In response, calves activate physiological and behavioral mechanisms as strategies to maintain homeostasis [3].

One key mechanism is an increase in respiratory rate, facilitating heat loss through evaporation, with rates rising by more than 50% in respiratory movements per minute [4,5,6]. This acceleration in gas exchange leads to increased carbon dioxide loss, altering the blood’s acid–base balance and causing blood alkalosis. To counterbalance blood alkalosis, the excretion of bicarbonate through urine increases [7]. Additionally, heart rate and blood pressure rise to compensate for peripheral vasodilation, increasing blood circulation to carry heat from internal organs to the body surface for dissipation [1].

Furthermore, water intake increases as a strategy to cool the body and replenish fluids through elevated respiratory activity and perspiration [8,9]. In adult animals, heat stress has been associated with increased insulin concentrations and decreased thyroid hormones due to altered cellular energy metabolism [10,11]. In calves, heat stress leads to reduced feed intake and performance, as a result of metabolic energy partitioning [12,13,14]. Heat stress also reduces gastrointestinal motility, alters ruminal microbiota, slows the passage rate, decreases digestibility, and impairs nutrient absorption due to diminished blood flow [1]. Moreover, in utero heat stress has been shown to have postnatal consequences, including reduced neonatal body and immune organ weights, increased jejunal enterocyte apoptosis, and delayed physical and immune system development [15]. Dairy calves subjected to early heat stress, even with post-pubertal weight gain recovery, exhibit a metabolic shift and fail to achieve optimal development [16].

Understanding the physiological and behavioral mechanisms of neonatal animals exposed to heat stress is crucial for developing management strategies and technologies to mitigate its impact. Nevertheless, few studies have evaluated these effects and their consequences on dairy calves under controlled temperature and humidity conditions [17,18]. Therefore, the objective of this study is to evaluate the effects of heat stress on Holstein dairy calves by assessing physiological variables, feed intake, ruminal parameters, nutrient digestibility, blood metabolites and hormones, health status, immune response, and performance during the first 28 days of life, using a climatic chamber with controlled environmental conditions. In addition, we assess the correlation between rectal temperature and infrared thermal images of the eye, flank, and perineum to explore the potential of non-invasive alternatives to traditional temperature measurement. We hypothesize that heat stress (1) increases the respiratory rate (RR), heart rate (HR), and rectal temperature (RT); (2) reduces feed intake and nutrient digestibility; (3) impairs growth and weight gain; and (4) negatively affects the health and immune responses in neonatal Holstein dairy calves.

## 2. Methods

Researchers conducted the study at the Experimental Farm of Embrapa Dairy Cattle, in the Multi-user Laboratory of Bioefficiency and Livestock Sustainability, in Coronel Pacheco, Minas Gerais, Brazil. All procedures involving animal care and handling were carried out in accordance with institutional and national guidelines and were approved by the Embrapa Dairy Cattle Animal Care and Use Committee (Protocol CEUA-EGL 4115231121). The experimental methods adhered to all applicable regulations and are reported in compliance with the ARRIVE guidelines [19].

### 2.1. Initial Care

Researchers monitored the dams throughout the dry period, beginning 60 days prior to calving. Between 60 and 30 days before parturition, cows were maintained on pasture with access to artificial shade, receiving a total mixed ration and unrestricted access to water. From 30 days prepartum, cows were transferred to a compost barn equipped with forced ventilation (average conditions: 23.1 ± 2.67 °C, 80.3 ± 4.13% relative humidity, wind speed 1.31 ± 0.32 m/s, and temperature–humidity index [THI] of 72 ± 4.19). Their diet was formulated with corn silage and concentrate to meet the nutrient requirements recommended by NASEM (2021) for late-gestation dairy cows [2].

A total of 34 Holstein calves (19 females and 15 males) were enrolled immediately after birth. The umbilical cords were treated with 10% iodine, and colostrum feeding was initiated within 2 h postnatally. Each calf received colostrum with a Brix value of 25% (equivalent to 10% of body weight), followed by an additional feeding of 5% of body weight 8 h later, administered via an oroesophageal tube. From days 2 to 4, calves were fed 6 L/day of transition milk at 37–39 °C divided into two meals (0730 and 1430 h) using via commercial milk bucked feeders (Milkbar, McInnes Manufacturing Limited, Waipu, New Zealand) inside the climatic chamber.

### 2.2. Animals, Facilities, and Environmental Temperature Control

Researchers conducted this study between January and October 2022 using 34 neonatal Holstein calves (19 females and 15 males). Calves were randomly assigned to two experimental groups after stratification by sex and birth weight to ensure a balanced distribution. The trial period spanned from birth until 28 days of age, during which calves were housed continuously in a climatic chamber. The treatments were as follows: control group (CON—11 females and 6 males) kept for 24 h at 22 °C and 65% humidity (THI of 66); and heat-stressed (HS—8 females and 9 males) group kept for 9 h (0630 to 1530 h) at 32 °C and 65% humidity (THI of 82), followed by 15 h (1531 to 0629 h) at 22 °C and 65% humidity (average THI of 66).

**Figure 1:**
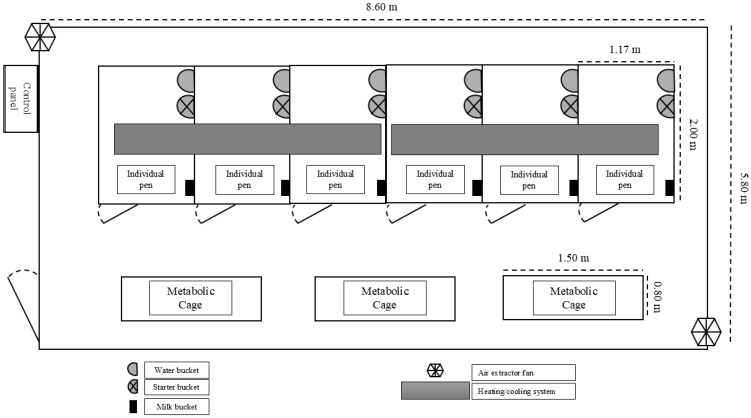
Open-circuit climatic chamber facilities.

Each calf was individually housed in a 2.00 × 1.17 m pen equipped with WingFlex^®^ rubber flooring (Kraiburg TPE GmbH & Co., Waldkraiburg, Germany) and wood shavings as bedding, within an open-circuit climatic chamber (Figure 1). Due to the single-chamber design, each treatment group was assessed in separate sequential blocks, alternating groups across the experimental timeline. Thermo-hygrometers (AK28 new, RS, Porto Alegre, Brazil) and maximum/minimum thermometers (FEPRO-MUT600S, Exbom, São Paulo, Brazil) were installed at calf height. Environmental temperature and relative humidity were recorded four times daily (0600, 1000, 1400, and 1600 h), coinciding with physiological data collection. These values were used to calculate the THI using the following equation [20], where RH represents the relative humidity and T is the temperature:

$$THI=\left(1.8\times T+32\right)-[\left(0.55-0.0055\times RH\right)\times \left(1.8\times T-26\right)]$$

### 2.3. Physiological Parameters

Researchers evaluated physiological parameters daily at 0600, 1000, 1400, and 1600 h. The respiratory rate (RR) was determined by counting thoracic-flank movements for 30 s and multiplying by two. The heart rate (HR) was assessed using a stethoscope placed on the left cardiac area, with beats counted for 30 s and multiplied by two. The rectal temperature (RT) was measured using a digital thermometer [21].

On days 7, 14, and 28 of age, thermal images were captured at each time point (0600, 1000, 1400, and 1600 h) using a thermal camera (FLIR T420; Systems Inc., Wilsonville, OR USA). Images were taken from a 1.5 m distance, targeting the left and right flanks, perineal region, and left and right eyes. Calves were restrained during imaging. Researchers analyzed images using FLIR TOOLS 5.6 software, identifying maximum surface temperatures based on thermal color mapping.

### 2.4. Diet, Intake, Performance, and Growth

Calves received 6 L/day of whole milk, divided into two meals (0730 and 1430 h), and had ad libitum access to starter feed and fresh water (Table 1). Researchers measured the daily individual intake by calculating the difference between the amount offered and the leftovers, using an electronic scale (Balmak, Santa Bárbara d’Oeste, Brazil). Milk refusals were measured with a graduated beaker. Feed efficiency was calculated as the ratio of average daily gain (ADG) to dry matter intake (DMI).

Researchers recorded body weight at birth and weekly thereafter using a mechanical scale (ICS 300, Coimma, Dracena, Brazil). Wither height, hip width, and chest girth were measured weekly using a portable height gauge (Walmur Veterinary Instruments Ltd., Porto Alegre, Brazil) and measuring tape on a level surface.

### 2.5. Ruminal Fermentation

On days 14 and 28, researchers collected ruminal fluid 3 h after the morning milk feeding using an oroesophageal tube. Samples were filtered through cotton gauze and the pH was measured immediately using a digital pH meter (T-1000, Tekna, Araucária, Brazil). Two 10 mL aliquots were taken: one acidified with 1 mL of 20% metaphosphoric acid and the other with 2 mL of 50% sulfuric acid. Both were stored at −20 °C for further analysis.

The ammonia nitrogen (NH_3_-N) concentration was determined using the colorimetric method of Chaney and Marbach (1962) [22]. Researchers measured absorbance at 630 nm using a spectrophotometer (Thermo Fisher Scientific, Madison, WI, USA) following Kjeldahl distillation. For the short-chain fatty acid (SCFA) analysis, samples were centrifuged at 1800× *g* for 10 min at room temperature (22–25 °C) and analyzed using high-performance liquid chromatography (Waters Alliance e2695 Chromatograph, Waters Technologies of Brazil LTDA, Barueri, SP, Brazil).

### 2.6. Digestibility

Researchers conducted two digestibility trials: the first from days 9 to 12, and the second from days 23 to 26. They removed the wood shavings from each pen to enable total feces collection. Composite fecal samples were collected over three consecutive days and stored at −20 °C. On the fourth day, calves were placed in metabolic cages (1.5 × 0.8 m; Ponta Ltd.a., Contagem, Brazil) for total urine collection. The total urine volume, weight, and density were recorded, and 50 mL aliquots were stored at −20 °C. Feed and refusals were also collected during this period and stored for subsequent analysis.

Researchers dried fecal, feed, and refusals samples in a forced-air oven at 55 °C for 72 h, then ground them using a Wiley mill (model 3, Arthur H. Thomas Co., Philadelphia, PA, USA) through a 1 mm mesh. Composite milk samples were collected daily and pooled weekly. These were lyophilized and ground for analysis.

Dry matter (DM; method 934.01), crude protein (CP; 988.05), ether extract (EE; 920.39), and organic matter (OM; 942.05) were analyzed following AOAC International (2012) [23]. The gross energy of feces, leftovers, the amount offered, and urine samples was determined using an adiabatic bomb calorimeter (IKA-C5000, IKA^®^ Works, Staufen, Germany). Apparent digestibility (%) was calculated using the amount of each component consumed and the amount recovered in the feces. The urinary nitrogen and energy were analyzed using the Kjeldahl method [23] and adiabatic bomb calorimeter (IKA-C5000, IKA^®^ Works, Staufen, Germany), respectively. The nitrogen balance was calculated as the difference between the dietary nitrogen intake and nitrogen excreted in feces and urine.

### 2.7. Blood Parameters

Researchers collected blood samples via jugular venipuncture 48 h after colostrum intake and again at 14 and 28 days of age, 3 h after the morning milk feeding. A sample collected on day 0 served as the baseline. The samples were used to determine the concentration of insulin, cortisol (collected in clot activator tube—code 11092, Labor Import, Osasco, Brazil), and glucose (collected in sodium fluoride tube—code 50213, Labor Import, Osasco, Brazil). All samples were centrifuged at 2500× *g* for 15 min and frozen at −20 °C for further analysis.

Serum cortisol and insulin concentrations were analyzed using chemiluminescence assays. Cortisol was measured using the ADVIA Centaur Cortisol kit (Immulite 2000 Systems 10381476, Cortisol 200, Siemens Healthcare Diagnostics Products Ltd., Llanberis, Gwynedd, UK), while insulin was analyzed with a Coat-a-Count kit (Immulite 2000 Systems 10381455, Insulin 200, Siemens Healthcare Diagnostics Products Ltd., Llanberis, Gwynedd, UK). Plasma glucose was assayed using the Glucose Plus Vet kit (ref. 90.068.00) by the colorimetric method, with an automatic analyzer (Cobas Mira Plus, Roche Diagnóstica Brazil Ltda., São Paulo, SP, Brazil).

On day 28, before feeding, researchers collected blood samples for cytokine analysis via jugular vein puncture (using tube without activator—Labor Import, Osasco, Brazil). These samples were analyzed for cytokines, including interferon-gamma (IFN-γ), interleukin 4 (IL-4), interleukin 8 (IL-8), interleukin 10 (IL-10), interleukin 17A (IL-17A), interferon-gamma-induced protein 10 (IP-10), macrophage chemotactic protein 1 (MCP-1), macrophage inflammatory protein 1 (MIP-1), and vascular endothelial growth factor (VEGF-A). Samples were stored at −20 °C until analysis, following the manufacturer’s instructions. The cytokines measurements were performed using a commercial kit (MILLIPLEX^®^ Bovine Cytokine/Chemokine kit, Millipore Corporation, Billerica, MA, USA) with antibodies against IFN-γ, IL-4, IL-8, IL-10, IL-17A, IP-10, MCP-1, MIP-1β, and VEGF-A.

### 2.8. Health Parameters

Fecal scores were assessed daily at 0600 h using the methodology adapted from McGuirk (2008) [24]. Calves were classified as having diarrhea when they presented fecal scores of 2 and 3, and severe diarrhea was considered when the fecal score was 3. Calves with diarrhea received two liters of oral rehydration solution (containing 10 g of NaCl, 12 g of sodium acetate, 2 g of KCl, and 40 g of glucose diluted in 2 L of water) twice daily, administered two h after feeding until clinical symptoms improved. Calves exhibiting both diarrhea and lethargy received oral rehydration solution and a single dose of anti-inflammatory medication (0.025 mL/kg, Maxicam 2%, Ouro Fino, Cravinhos, Brazil). The total volume of oral rehydration solutions administered per calf, as well as the dosage of anti-inflammatory medication was recorded. Days with hyperthermia were defined as those when the calf’s body temperature exceeded 39.4 °C, while days with severe hyperthermia were defined as those with a temperature above 41.0 °C [21].

### 2.9. Statistical Analysis

Seventeen experimental units were assigned to each treatment. The collected data were divided into four weeks: week 1 (0 to 7 days), week 2 (8 to 14 days), week 3 (15 to 21 days), and week 4 (22 to 28 days), and analyzed using R software (R Core Team, 2024, version 4.3.3) [25].

A linear mixed-effects model was used to evaluate independent variables such as intake, performance, clinical parameters, blood, and ruminal parameters (nlme package). The model included the fixed effects of treatment, week (or time for clinical parameters), and their interaction. The calf was included as a random effect to account for repeated measures within animals. Birth weight, the BRIX value of passive immunity transfer, and sex were tested as covariates and included in the model when significant (*p* < 0.05). Since calves from the same treatment group were housed together in the climatic chamber during the same period, the time period during which each treatment was conducted was included as a random effect. Digestibility, nitrogen balance, days in diarrhea/hyperthermia, and cytokine analysis were evaluated using ANOVA (R Core Team, 2024, version 4.3.3), where the treatment was the fixed effect, and animals and period were the random effects, employing a generalized linear model. For cytokine analyses, day 0 values were included as covariates. All models were tested for normality and homoscedasticity using the Shapiro–Wilk and Bartlett tests. A 95% confidence interval was employed to assess the null hypothesis, and *p*-values were determined using the Tukey test.

Spearman’s correlation analysis on residuals was conducted on the eye, flank, perineal area, and rectal temperature. Correlation values between ± 0.5 and ± 1.0 were considered strongly correlated, values between ± 0.30 and ± 0.49 moderately correlated, and values between 0 and ± 0.29 lowly correlated. Outcomes such as fecal scores were analyzed using a ranking test performed with the ARTool package (R Core Team, 2024, version 4.3.3).

## 3. Results

### 3.1. Intake and Physiological Parameters

**Figure 2:**
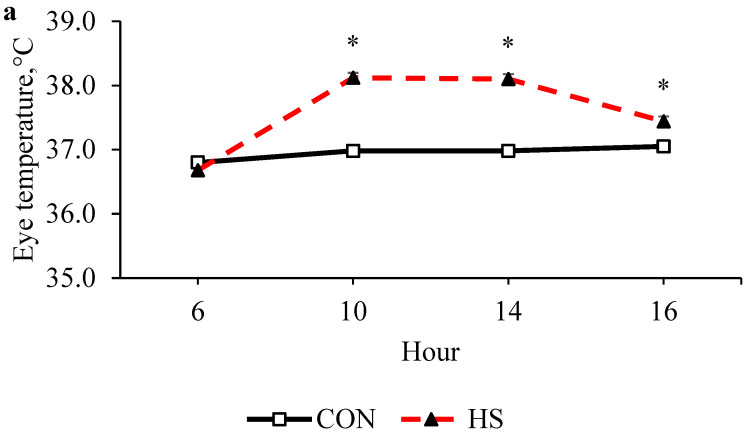

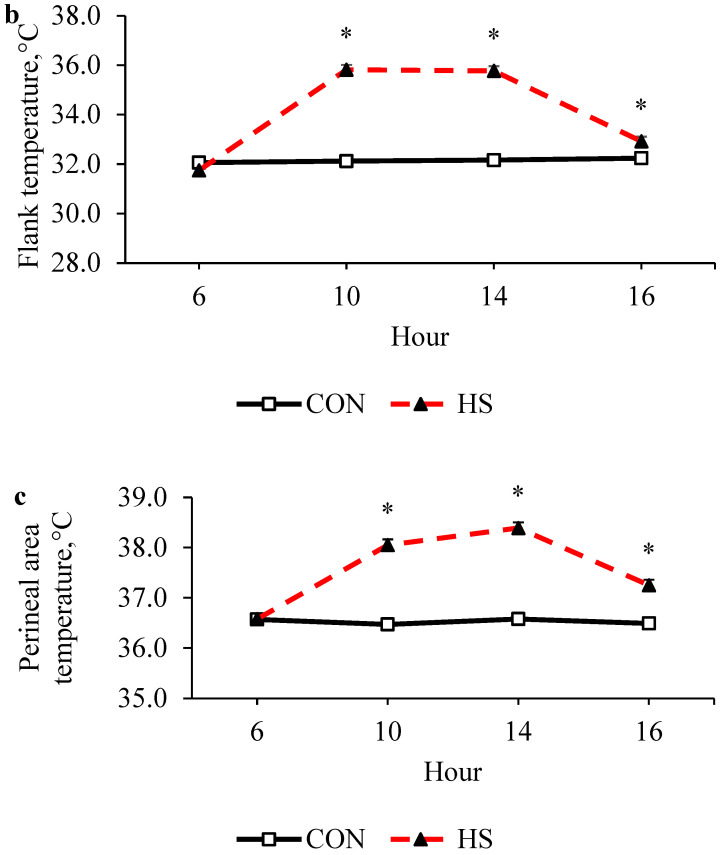
Surface temperatures of eye, flank, and perineal temperature of control and heat-stressed Holstein calves. (**Panel a**): Effect of treatment (*p* < 0.001); effect of hour (*p* < 0.01); effect of treatment–hour interaction (*p* < 0.001). (**Panel b**): Effect of treatment (*p* < 0.001); effect of hour (*p* < 0.001); effect of treatment–hour interaction (*p* < 0.001). (**Panel c**): Effect of treatment (*p* < 0.001); effect of hour (*p* < 0.001); effect of treatment–hour interaction (*p* < 0.001). Error bars indicate SEM. Asterisk denotes interaction.

**Figure 3:**
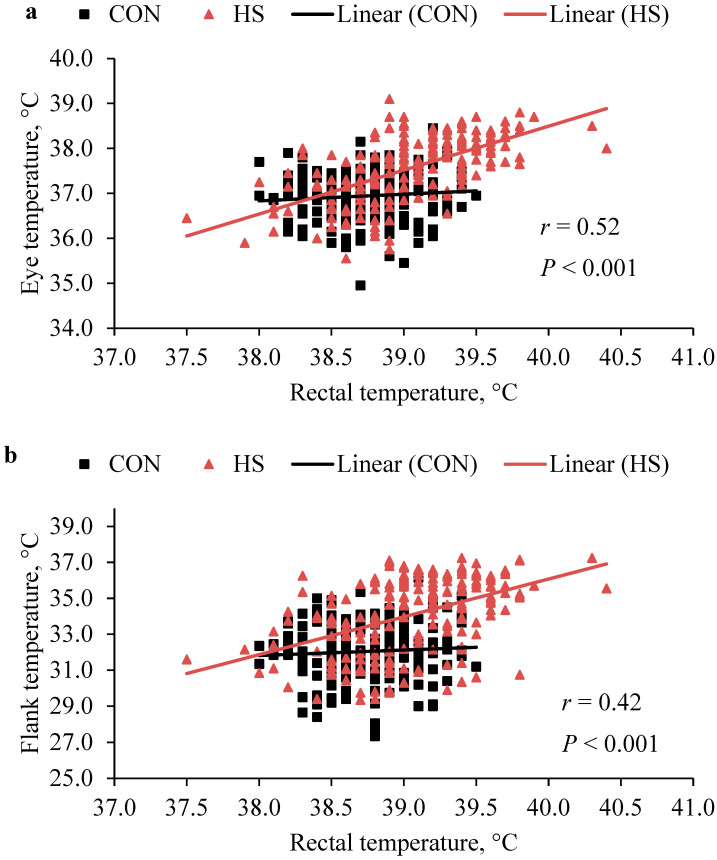

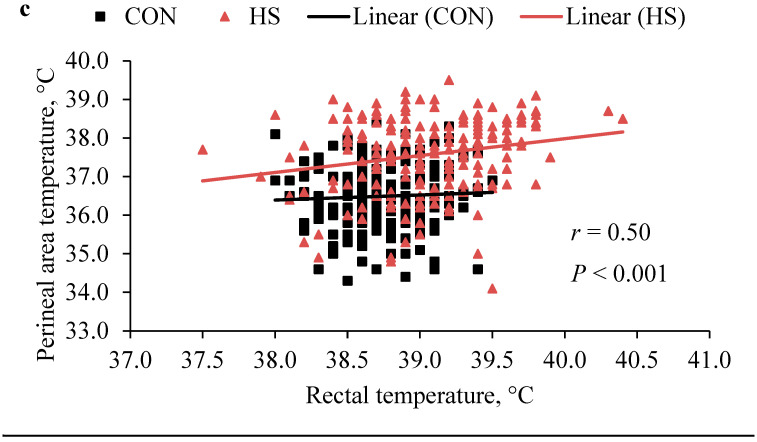
(**Panel a**): Correlation between eye temperature measurement and rectal temperature. (**Panel b**): Correlation between flank temperature and rectal temperature. (**Panel c**): Correlation between perineal area temperature and rectal temperature. Surface temperature images of the eye, flank, and perineal area were taken using a thermal camera model FLIR T420 portable device (FLIR Systems Inc., Wilsonville, OR, USA).

The HS calves had greater RR and RT at 1000, 1400, and 1600 h (*p* < 0.01; Table 2, Figure 2). The effect of week was also significant for all parameters evaluated, with higher RR and HR observed in weeks 1 and 2, and higher rectal temperatures in 2 and 3 (*p* < 0.001, Table 2). When measured with the thermal camera, there was a moderate correlation with RT (Figure 3). Surface temperatures of the eye, flank, and perineal regions differed significantly between CON and HS (Figure 2) and were positively correlated with rectal temperature (Figure 3).

Milk, starter, CP, and DM intakes did not differ between treatments (*p* > 0.05, Table 3), but increased with advancing age (*p* < 0.001; Table 3). The water intake in the HS treatment was 59.5% higher (*p* < 0.03; Table 3), with differences of 0.67, 0.79, 1.46, and 1.54 L in weeks 1, 2, 3, and 4, respectively, compared to the CON treatment (*p* < 0.05).

Initial and final weights, average daily gain (ADG), feed efficiency, rump width, and withers height were not affected by HS (*p* > 0.05, Table 3). However, chest girth decreased in the HS treatment (*p* = 0.03; Table 3). These responses increased with the age of the animals, showing differences among weeks (*p* < 0.05; Table 3).

### 3.2. Ruminal Fermentation and Blood Metabolites

HS decreased acetate and propionate concentrations (*p* < 0.05; Table 3). However, pH, NH_3_-N, butyrate, and the acetate-to-propionate ratio were not influenced by HS (*p* > 0.05; Table 4). A significant week effect was observed for propionate and butyrate concentration, as well as for the acetate-to-propionate ratio, with values increasing as the animals aged (*p* < 0.05; Table 4). HS did not affect the concentration of blood metabolites or hormones. A significant week effect was noted, with glucose values decreasing and the concentrations of other metabolites and hormones increasing as the animals grew older (*p* < 0.001; Table 4).

### 3.3. Digestibility

Heat-stressed reduced the digestibility of EE in both digestibility 1 and 2 and decreased on 49.4% the urinary nitrogen in digestibility 1 (*p* < 0.05; Table 5). The digestibility of DM, OM, CP, and GE, nitrogen intake, fecal nitrogen, urinary nitrogen, retained nitrogen, and urine volume were not different between treatments.

### 3.4. Blood Cytokine

For the measured cytokines, the HS treatment increased values of IL-4 and decreased values of IL-8 and IP-10 (*p* < 0.001; Table 6).

### 3.5. Health Parameters

The fecal score and days with diarrhea and severe diarrhea did not differ among treatments (*p* > 0.05, Table 7). However, calves in the HS treatment experienced 12.78 additional days with hyperthermia, and an 85.9% increase in days with severe hyperthermia than the CON treatment (*p* < 0.001, Table 7). There was no significant difference in the number of days calves received an oral rehydration solution between treatments (7.0 ± 1.1 days for CON treatment and 5.4 ± 0.9 days for HS treatment, *p* = 0.28).

## 4. Discussion

This study aimed to mimic the effect of heat stress on neonatal dairy calves and assess its impact on physiological responses, water and nutrient intake, ruminal fermentation, nutrient digestibility, serum glucose and hormone concentrations, cytokine profiles, health parameters, and overall performance. To our knowledge, this is one of the first research studies to comprehensively evaluate neonatal dairy calves in a fully controlled thermal environment during the first month of life.

We hypothesized that neonatal heat stress would (1) increase the respiratory rate, heart rate, and rectal temperature; (2) reduce nutrient intake and digestibility; (3) impair growth and body development; and (4) alter inflammatory and hormonal responses. These hypotheses were partially confirmed. Heat-stressed calves exhibited clear physiological adaptations and immune alterations, with limited effects on performance. The main findings were as follows: calves stressed by heat (i) showed greater respiratory rates and rectal temperatures; (ii) consumed more water and maintained similar urinary output; (iii) exhibited altered ruminal fermentation patterns; (iv) had lower ether extract (EE) digestibility in both digestibility trials and greater urinary nitrogen in the first digestibility trial; (v) had greater values of anti-inflammatory cytokines and lower values of chemokines and pro-inflammatory cytokines; and (vi) showed a smaller thoracic circumference but did not show differences in ADG.

Calves are considered more heat-tolerant than older cattle due to their relatively larger body surface area compared to internal mass, which facilitates heat exchange with the environment [1]. As pre-ruminants, ruminal fermentation becomes more prominent starting in the third week of life, following increased solid diet intake [26]. Consequently, neonatal calves produce less heat from ruminal fermentation than adults, given that their diet is primarily liquid during the first two months of life.

The normal rectal temperature range for cattle is 38 to 39.4 °C [27], and the values observed in both treatments fell within this range. However, the HS calves had an average RT that was 0.4 °C higher than the CON calves. When cattle are exposed to heat, they rapidly increase rectal temperature, thereby enhancing the temperature gradient between their skin surface and the ambient environment [21]. Therefore, body temperature rises in response to ambient conditions, and RR also increases as an adaptive mechanism to promote heat loss [28].

An elevated RR draws external air to the alveoli, where it is warmed to body temperature. During exhalation, this heated air cools as it passes over the mucosa, releasing body heat through condensation [29], resulting in up to 20% of evaporative losses [30]. Although both RR and RT increase in response to higher ambient temperature, HR did not differ between treatments and remained within the normal range (90 to 160 beats/min [30]), consistent with previous observations in neonatal calves subjected to heat stress or cooling [31].

The respiratory process also results in body water loss and likely contributed to the 210% increase in water intake observed among HS calves during their first four weeks of life. While greater water intake typically leads to increased excretion in animals within the thermoneutral zone, urinary volume did not increase in the HS calves [32]. This suggests that the excess water was likely utilized in evaporative processes such as panting and sweating [33], rather than excreted.

Heat production from roughage fermentation in the rumen of adult animals is a key factor contributing to reduced feed intake under high temperatures. In contrast, calves in their first month of life mainly consume milk, with only a small proportion of starter intake [34]. Therefore, they generate less heat from ruminal fermentation, and may not experience major reductions in liquid diet intake [31], but can present a decrease in starter intake [28]. In this study, the main difference in nutrient intake was a daily increase of about 1087 g of water in HS calves compared to CON calves. Since nutrient intake depends on physiological parameters and saliva production, shifts in these factors can alter the ruminal environment and fermentation patterns, often reducing acetate concentrations in heat-stressed animals [35]. This aligns with our observation that acetate and propionate were lower in heat-stressed calves, as was EE digestibility in both digestibility trials. Although greater water intake may speed up feed passage and reduce nutrient breakdown, previous studies have shown inconsistent results on whole-tract digestibility, with some finding no differences [36], or lower day matter, organic matter, crude protein, and ether extract digestibility [37]. In this study, reduced EE digestibility in both trials may be linked to faster RR and panting, which could interfere with saliva swallowing and decrease the effects of salivary lipase on short-chain fatty acids in milk. Also, because overall nutrient intake did not differ between treatments, feed efficiency remained similar, corroborating findings from Holstein dairy cows in climatic chambers [38], and female Holstein calves housed in a barn without cooling [39].

Altered physiology and nutrient digestibility may explain the smaller thoracic circumference in HS animals. Their higher urinary nitrogen excretion in the first digestibility trial suggests possible protein catabolism to supply nutrients for immune function or other processes demanded by heat adaptation, diverting resources from growth. Since calves achieve roughly 25% of their growth in the first six months of life [40], such variations could affect the final body frame and, in turn, future productivity. Despite these differences in morphology, HS calves did not show reduced ADG, indicating that the moderate milk supply of 6 L per day (approximately 3.26 Mcal ME/d) was sufficient to cover elevated maintenance requirements for calves that started at 35 kg and reached roughly 50 kg at 28 days of age, with gains of 500 to 600 g/d [2].

Although glucose is an important energy source for animals under heat stress, we found no difference in serum glucose concentrations between the HS and CON treatments during the weeks evaluated. An inflammatory response might have been expected to lower serum glucose as phagocytosis increases glucose use [41], and daily glucose requirements typically rise in sick animals [42]. However, 6 L of milk, rich in lactose, a glucose precursor, likely satisfied the energy needs of both treatments. In addition, both treatments received glucose-containing electrolytes if neonatal diarrhea occurred, possibly further supporting glucose homeostasis during diarrhea. Heat stress can also modulate other hormones and metabolites, such as cortisol, which initially rises with acute heat exposure and then normalizes or decreases during prolonged stress [43,44,45]. However, in this study, no differences were observed in any of the measured metabolites or hormones.

By contrast, HS calves experienced more days with hyperthermia, more days with severe hyperthermia, and higher levels of the anti-inflammatory cytokine IL-4, alongside lower concentrations of pro-inflammatory cytokines IL-8 and IP-10. The IL-4 increase may represent a protective response to minimize heat-induced cellular damage, assisting tissue repair and reducing inflammation through a Th2-type immune mechanism [11]. The decrease in IL-8 and IP-10 may have reduced the recruitment and activity of innate immune cells, potentially lowering the overall inflammatory burden [46], and affecting the animal’s microbiome, rumen colonization, nutrient digestibility, and conversion of nutrients into muscle and bone. Though not measured here, such effects on the microbiome could help explain morphometric and metabolic changes.

Rectal temperature remains a standard tool for identifying heat stress in cattle [47], but due to labor constraints, the infrared thermography of body surfaces could represent a useful non-invasive alternative. In this study, eye temperature showed a moderate correlation with rectal temperature, but a consistent distance from the animal and control of external factors like bedding moisture are critical to ensure accurate readings [48].

Although this study focused on the first month of life, a critical period for immune development and thermoregulation, it did not assess long-term outcomes such as changes in organ weights, body composition, or subsequent lactation performance. Future research should explore these long-term effects to fully understand the implications of early-life heat stress.

## 5. Conclusions

Calves exposed to a THI of 82 for 9 h exhibit altered physiological parameters, increased water intake, reduced EE digestibility, and changes in ruminal fermentation patterns and nutrient digestibility, without compromising average daily gain or feed efficiency. Their immune response shifts toward a higher anti-inflammatory and lower pro-inflammatory cytokine profile, while hormones and metabolite levels remain largely unaffected. Although they present a slightly smaller thoracic circumference, these calves demonstrate resilience to moderate heat stress during the first month of life, underscoring the importance of adequate nutrition and supportive management during this critical developmental stage.

## Figures and Tables

**Table 1:** Nutritional composition (DM basis) of milk and starter. ^1^ DM = dry matter; CP = crude protein; EE = ether extract; GE = gross energy. ^2^ Starter = corn, soybean meal, mineral supplement.

Nutrient ^1^	Whole Milk	Starter ^2^
DM (%)	12.66	86.02
CP (% of DM)	21.90	19.20
EE (% of DM)	27.60	2.50
NDF (% of DM)	-	9.18
Ash (% of DM)	5.45	8.30
Lactose (%)	45.05	-
GE (Mcal/kg)	5.54	4.05

**Table 2:** Respiratory rate (RR), heart rate (HR), and rectal temperature (RT) of calves from the control treatment (CON) and the heat-stressed treatment (HS). ^1^ Item: RR = respiratory rate; HR = heart rate; RT = rectal temperature. ^2^ Treatment: CON = control (n = 17 calves); HS = heat-stressed (n = 17 calves); ^3^ SEM = standard error of the mean; ^4^ T = treatment; H = hour; W = week; T × H = treatment–hour interaction; T × W = treatment–week interaction; significant when *p* < 0.05.

	Treatment ^2^			*p*-Value ^4^				
Item ^1^	CON	HS	SEM ^3^	T	H	W	T × H	T × W
RR, mov/min	36.88	57.93	8.25	<0.001	<0.001	0.001	<0.001	<0.001
HR, beats/min	132.52	135.60	14.61	0.28	<0.001	<0.001	0.48	<0.001
RT, °C	38.74	39.03	0.20	<0.001	<0.001	<0.001	<0.001	0.01

**Table 3:** Milk, starter, and water intake, calf performance, in the control (CON) and heat-stressed (HS) treatments from 0 to 28 days of age. ^1^ Treatment: CON = control (n = 17 calves); HS = heat-stressed (n = 17 calves). ^2^ SEM = standard error of the mean. ^3^ T = treatment; W = week; T × W = treatment–week interaction. ^4^ DM = dry matter. ^5^ CP = crude protein. ^6^ ADG = average daily gain.

Item	Treatments ^1^			*p*-Value ^3^		
	CON	HS	SEM ^2^	T	W	T × W
Intake						
Milk (g of DM/d)	727.02	715.89	41.1	0.18	<0.001	0.38
Starter (g of DM/d)	39.21	27.20	28.1	0.08	<0.001	0.84
Total DM ^4^ (g of DM/d)	783.35	771.40	5.33	0.31	<0.001	0.96
Total CP ^5^ (g/d)	166.51	163.45	9.55	0.19	<0.001	0.78
Water (kg/d)	0.740	1.827	0.48	<0.001	<0.001	0.03
Performance						
Initial weight (kg)	35.65	35.93	0.37	0.85	-	-
Final weight (kg)	51.94	51.90	0.00007	0.97	-	-
ADG ^6^ (g/d)	488.12	557.80	195.0	0.10	<0.001	0.48
Withers height (cm)	79.93	79.89	1.06	0.92	<0.001	0.24
Rump width (cm)	23.26	22.82	0.95	0.10	<0.001	0.79
Chest circumference (cm)	83.07	81.74	1.17	0.03	<0.001	0.89

**Table 4:** Ruminal concentration of short-chain fatty acids (SCFAs), pH, and ruminal ammonia nitrogen (NH_3_-N) and blood metabolites of calves in the control (CON) and heat-stressed (HS) treatments. ^1^ Treatment: CON = control (n = 17 calves); HS = heat-stressed (n = 17 calves); ^2^ SEM = standard error of the mean. ^3^ T = treatment; W = week; T × W = treatment–week interaction. Significant when *p* < 0.05. ^4^ NH_3_-N = ammonia nitrogen.

	Treatments ^1^			*p*-Value ^3^		
Item	CON	HS	SEM ^2^	T	W	T × W
Rumen						
pH	6.36	6.39	0.58	0.67	0.12	0.21
NH_3_-N ^4^ (%)	13.11	9.87	4.52	0.13	0.29	0.09
Acetate (µmol/L)	20.11	13.63	8.6	0.009	0.30	0.27
Propionate (µmol/L)	10.01	5.61	4.7	0.01	<0.001	0.64
Butyrate (µmol/L)	3.64	2.89	1.53	0.19	0.02	0.12
Acetate–propionate ratio	2.60	2.28	0.0002	0.19	<0.01	0.63
Blood						
Glucose (mg/dL)	124.45	127.59	16.5	0.32	<0.001	0.27
Albumin (mg/dL)	1.42	1.49	0.38	0.56	<0.001	<0.001
Cholesterol (mg/dL)	24.60	25.04	9.70	0.96	<0.001	0.28
Creatinine (mg/dL)	0.47	0.47	0.08	0.90	<0.001	0.01
Total Protein	1.91	1.92	0.22	0.99	<0.001	0.30
Triglycerides	9.08	10.72	3.63	0.18	<0.001	0.77
Insulin (ng/mL)	11.34	13.29	14.9	0.41	0.006	0.09
Cortisol (µg/dL)	0.53	0.57	0.25	0.49	0.01	0.19

**Table 5:** Apparent digestibility of nutrients (%) and nitrogen balance in calves from the control (CON) and heat-stressed (HS) treatment between 9 and 12 days of age (Digestibility 1) and between 23 and 26 days of age (Digestibility 2). ^1^ Item: DM = dry matter; OM = organic matter; CP = crude protein; EE = ether extract; GE = gross energy. ^2^ Treatment: CON = control (n = 10 calves); HS = heat-stressed (n = 10 calves). ^3^ SEM = standard error of the mean. Significant when *p* < 0.05.

	Treatments ^2^			*p*-Value
Item ^1^	CON	HS	SEM ^3^	
Digestibility 1				
DM (%)	96.04	94.46	0.15	0.12
OM (%)	99.57	99.35	0.008	0.10
CP (%)	92.13	89.42	0.05	0.21
EE (%)	97.09	90.36	0.31	0.04
GE (%)	99.45	99.28	0.02	0.08
N intake (g/d)	29.43	30.04	0.00003	0.63
Fecal N (g/d)	2.15	2.86	0.04	0.17
Urinary N (g/d)	3.06	6.04	0.00002	0.02
Retained N (g/d)	23.47	20.35	0.00004	0.13
Urine volume (L/d)	3.21	3.32	0.07	0.73
Digestibility 2				
DM (%)	96.81	94.91	0.20	0.10
OM (%)	99.60	99.35	0.03	0.09
CP (%)	93.46	90.89	0.47	0.28
EE (%)	97.40	93.53	0.30	0.03
GE (%)	99.55	99.26	0.03	0.08
N intake (g/d)	30.74	32.16	0.17	0.19
Fecal N (g/d)	1.94	2.92	0.00006	0.28
Urinary N (g/d)	5.27	5.37	0.27	0.93
Retained N (g/d)	22.93	23.23	0.45	0.91
Urine volume (L/d)	3.70	3.87	0.000009	0.69

**Table 6:** Blood cytokine concentrations of calves in control (CON) and heat-stressed calves (HS) treatments at 28 days of age. ^1^ IFN = interferon gamma, IL = interleukin, IP = interferon gamma-induced protein, MCP = monocyte chemoattractant protein, MIP = macrophage inflammatory protein, VEGFA = vascular endothelial growth factor A. ^2^ Treatment: CON = control (n = 8 calves); HS = heat-stressed (n = 8 calves). ^3^ SEM = standard error of the mean. Significant when *p* < 0.05.

	Treatments ^2^			
Item ^1^	CON	HS	SEM ^3^	*p*-Value
IFN (pg/mL)	4.33	7.65	0.29	0.18
IL-4 (pg/mL)	27.03	41.31	0.42	<0.001
IL-8 (pg/mL)	125.08	80.25	0.58	<0.001
IL-10 (pg/mL)	13.62	6.65	0.45	0.09
IL-17A (pg/mL)	1.26	0.95	0.11	0.59
IP-10 (pg/mL)	6789.86	999.28	206	<0.001
MCP-1 (pg/mL)	213.62	156.81	25.97	0.32
MIP-1 (pg/mL)	72.81	31.99	0.005	0.22
VEGFA (pg/mL)	12.44	14.99	0.60	0.69

**Table 7:** Health score of calves in the control (CON) and heat-stressed (HS) treatments. ^1^ Treatment: CON = control (n = 17 calves); HS = heat-stressed (n = 17 calves); ^2^ SEM = standard error of the mean. ^3^ T = treatment. W = week; T × W = treatment–week interaction. Significant when *p* < 0.05.

	Treatments ^1^				*p*-Value ^2^	
Item	CON	HS	SEM	T ^3^	W	T × W
Fecal score	0.57	0.59	0.03	0.16	<0.001	<0.001
Days in diarrhea	7.56	5.17	0.05	0.10	-	-
Days with severe diarrhea	5.23	3.53	0.19	0.15	-	-
Days with hyperthermia	2.85	15.6	0.27	<0.001	-	-
Days with severe hyperthermia	0.14	0.99	0.04	<0.001	-	-

## Data Availability

The datasets from the current study are available from the corresponding author upon reasonable request.
